# The Effect of Histological Subtypes on Outcomes of Stage IV Epithelial Ovarian Cancer

**DOI:** 10.3389/fonc.2018.00577

**Published:** 2018-12-04

**Authors:** Juan Zhou, San-Gang Wu, Jun Wang, Jia-Yuan Sun, Zhen-Yu He, Xin Jin, Wen-Wen Zhang

**Affiliations:** ^1^Department of Obstetrics and Gynecology, The First Affiliated Hospital of Xiamen University, Xiamen, China; ^2^Department of Radiation Oncology, Xiamen Cancer Hospital, The First Affiliated Hospital of Xiamen University, Xiamen, China; ^3^State Key Laboratory of Oncology in South China, Collaborative Innovation Center of Cancer Medicine, Department of Radiation Oncology, Sun Yat-sen University Cancer Center, Guangzhou, China; ^4^Department of Basic Medical Science, Medical College, Xiamen University, Xiamen, China

**Keywords:** ovarian neoplasms, histology, survival analysis, neoplasm, residual, gynecologic surgical procedures

## Abstract

**Introduction:** To examined survival outcome by histological subtypes in *de novo* stage IV epithelial ovarian cancer (EOC).

**Methods:** Between 2004 and 2015, patients with stage IV EOC were included using the Surveillance, Epidemiology, and End Results program. The effects of histological subtypes on overall survival (OS) were assessed using Kaplan–Meier and multivariable Cox regression analyses.

**Results:** We identified 5,953 patients including 5,351 (89.9%), 249 (4.2%), 145 (2.4%), and 208 (3.4%) patients with high-grade serous, endometrioid, mucinous, and clear cell subtypes, respectively. The 5-year OS rates were 28.1, 38.6, 14.2, and 18.8% in patients with high-grade serous, endometrioid, mucinous, and 18.8% clear cell subtypes, respectively, (*p* < 0.001). Multivariate analyses indicated that histological subtype was an independent predictor of OS. Using the high-grade serous subtype as a reference, OS was comparable for the endometrioid subtype (hazard ratio (HR) 0.915, 95% confidence interval) (CI 0.772–1.085, *p* = 0.305), but significantly lower for mucinous (HR 3.292, 95% CI 2.701–4.011, *p* < 0.001) and clear cell subtypes (HR 1.820, 95% CI 1.546–2.141, *p* < 0.001). Patients with no residual tumor had better OS in the high-grade serous and endometrioid subtypes compared to patients with residual tumors. However, the residual tumor size was not a prognostic factor for OS in mucinous and clear cell carcinoma.

**Conclusions:** Our study suggest a markedly mortality rate in patients with stage IV mucinous and clear cell carcinoma, but better survival in patients with high-grade serous and endometrioid subtypes. Aggressive radical surgery to leave no residual disease would improve survival for high-grade serous and endometrioid carcinoma. More studies are needed to assess the value of aggressive radical surgery in patients with mucinous and clear cell subtypes.

## Background

Epithelial ovarian cancer (EOC) is the most lethal gynecologic malignancy (1). Due to lack of obvious and specific symptoms, ~80% of EOC were diagnosed at an advanced stage, and 28% of them present with distant metastasis (stage IV disease) (2–4). The standard treatment for advanced EOC is primary surgery aiming at a complete resection, followed by platinum and taxane-based chemotherapy (5). However, the 5-year cause-specific survival is only 20% for stage IV EOC (2). The performance status, the presence of residual tumors, metastatic sites, and no debulking surgery are known prognostic factors affecting the survival of patients with stage IV EOC (6–8).

Five following histological subtypes of EOC were distinguished in 2014: low-grade serous, high-grade serous, endometrioid, mucinous, and clear cell carcinoma (9). However, few studies have focused specifically on patients with stage IV EOC. In addition, there were several limitations in previous studies that focused on histotype-specific survival patterns. The majority of studies examining survival of EOC by histological subtypes did not delineate the specificity of the histological subtypes, but only used serous and non-serous subtypes to compare the survival outcomes of patients (6–8). Given that the current specificity of histological subtypes may more accurately reflect the survival of patients with EOC, in the present study, we evaluated survival patterns by histological subtypes using a population-based cancer registry.

## Materials and Methods

### Patients

The Surveillance, Epidemiology, and End Results (SEER) 18 population-based cancer registries were used in this study. We obtained permission to access the SEER database with authorization code 11025-Nov2016. The de-identified information of patients including demographic, clinicopathological characteristics, first course of treatment, and vital status were included. Patients with EOC, including high-grade serous, mucinous, endometrioid, and clear cell carcinoma, who had undergone surgery and chemotherapy between 2004 and 2015, were identified in this study^1^. We excluded patients with uncommon EOC, such as low-grade serous carcinoma, carcinosarcoma, malignant Brenner carcinoma, and mixed subtypes. Patients without positive histology for EOC were also excluded. Using data from SEER was exempt from the approval process of Institutional Review Boards because of the de-identified information of the patients.

### Variables

Demographic, clinicopathological, and treatment variables were included as follows: age, race/ethnicity, grade, nodal status, histocytes, metastatic site, and residual tumor size (after 2010). The histological subtypes were classified as high-grade serous, endometrioid, mucinous, and clear cell. The metastatic site was defined as the code “CS Mets at DX” of the SEER program, including distant lymph node only (code 10), liver parenchymal metastasis or pleural effusion with positive cytology (code 40), and code 40+10. After 2010, data on the size of the residual disease after primary cytoreduction surgery were included in the SEER program, and the classification of the size of residual disease was as follows: no residual tumor, ≤1 cm residual tumor, and >1 cm residual tumor.

### Statistical Analysis

The Chi-squared test and Fisher exact probability tests were used to compare the frequencies of the patient demographic and clinicopathological variables among the histological subtypes. Kaplan–Meier analyses for 5-year overall survival (OS) were performed and compared using a log-rank test. OS was defined as the date of diagnosis until the date of death or last follow-up. A Cox proportional hazards model was used for multivariate analyses. All analyses were performed using SPSS version 22 statistical software (IBM Corporation, Armonk, NY, USA), and a *p* < 0.05 was considered significant.

## Results

### Patients' Clinicopathological Data

We identified 5,953 patients with stage IV EOC including 5,351 (89.9%), 249 (4.2%), 145 (2.4%), and 208 (3.4%) patients with high-grade serous, endometrioid, mucinous and clear cell subtypes, respectively, (Table 1). A total of 76.6% (*n* = 4,561) of patients had liver parenchymal metastasis or positive cytology in their pleural effusion. Patients with the serous subtype were more likely to be older (*p* < 0.001), non-Hispanic White (*p* = 0.001), poorly/undifferentiated disease (*p* < 0.001), and regional (*p* < 0.001) and distant (*p* < 0.001) lymph node metastasis.

**Table 1:** Baseline characteristics of epithelial ovarian cancer patients by histologic subtypes.

**Variables**	***n***	**High-grade serous (%)**	**Endometrioid (%)**	**Mucinous (%)**	**Clear cell (%)**	***p***
**AGE (YEARS)**						
<60	2,542	2,175 (40.6)	145 (58.2)	97 (66.9)	125 (60.0)	<0.001
≥60	3,411	3,176 (59.4)	104 (41.8)	48 (33.1)	83 (39.9)	
**RACE/ETHNICITY**						
Non-hispanic white	4,329	3,929 (73.4)	163 (65.5)	96 (66.2)	141 (67.8)	0.001
Non-hispanic black	431	386 (7.2)	21 (8.4)	16 (11.0)	8 (3.8)	
Hispanic	688	603 (11.3)	38 (15.3)	19 (13.1)	28 (13.5)	
Other	505	433 (8.1)	27 (10.8)	14 (9.7)	31 (14.9)	
**GRADE**						
Well-differentiated	38	0 (0)	13 (5.2)	24 (16.6)	1 (0.5)	<0.001
Moderately differentiated	528	423 (7.9)	59 (23.7)	34 (23.4)	12 (5.8)	
Poorly/undifferentiated	4,179	3,866 (72.2)	147 (59.0)	49 (33.8)	117 (56.3)	
Unknown	1,208	1,062 (19.8)	30 (12.0)	38 (26.2)	78 (37.5)	
**NODAL STATUS**						
Negative	3,029	2,685 (51.2)	141 (56.6)	90 (62.1)	113 (54.3)	<0.001
Positive	2,198	2,020 (37.8)	72 (28.9)	31 (21.4)	75 (36.1)	
Unknown	726	646 (12.1)	36 (14.5)	24 (16.6)	20 (9.6)	
**METASTATIC SITE**						
Distant lymph node only (A)	512	484 (9.0)	16 (6.4)	3 (2.1)	9 (4.3)	<0.001
Liver parenchymal metastasis or pleural effusion with positive cytology (B)	4,561	4,053 (75.7)	209 (83.9)	129 (89.0)	170 (81.7)	
A+B	577	541 (10.1)	14 (5.6)	8 (5.5)	14 (6.7)	
Other	303	273 (5.1)	10 (4.0)	5 (3.4)	15 (7.2)	
**RESIDUAL TUMOR (*****n*** = **1,612)**						
No	799	720 (48.9)	38 (58.5)	13 (61.9)	28 (50.9)	0.166
≤1 cm	490	460 (31.3)	14 (21.5)	2 (9.5)	14 (25.5)	
>1 cm	323	291 (19.8)	13 (20.0)	6 (28.6)	13 (23.6)	

A total of 1,612 patients had recorded details of residual tumors after surgery. Approximately half of the patients had no residual tumor. The residual tumor size was not significantly different among the four histological subtypes (*p* = 0.166).

### Survival and Prognostic Analysis

**Figure 1:**
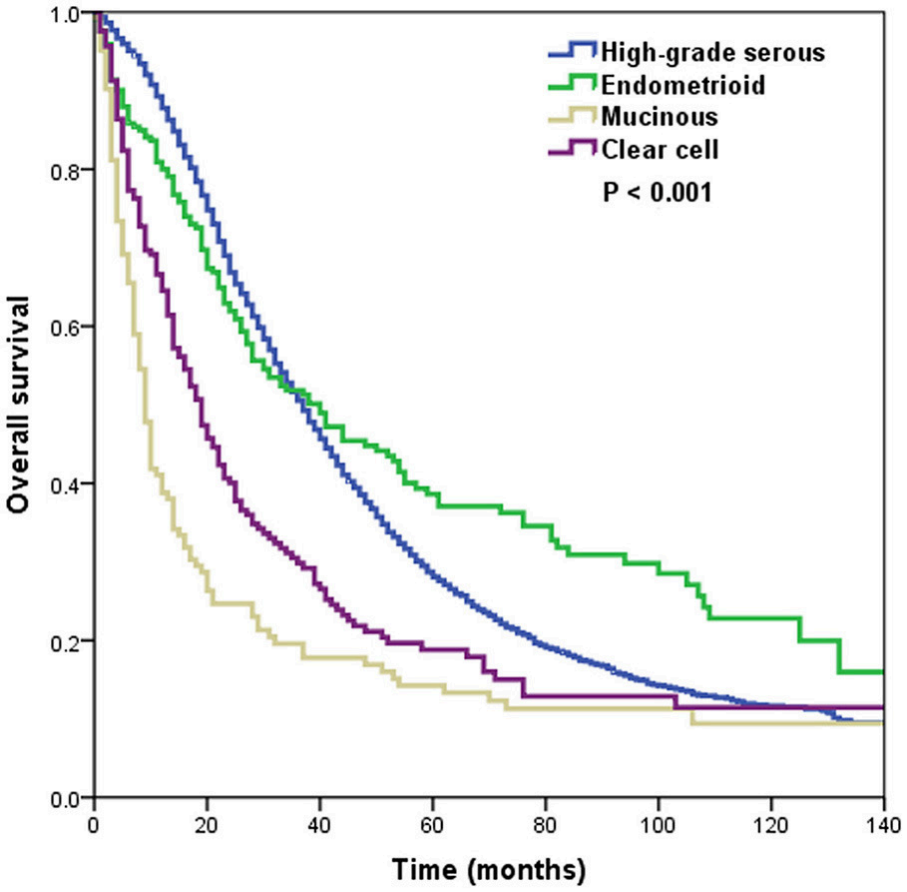
Overall survival by histological subtypes.

A total of 3,709 deaths were recorded during the study period, and most of them were died with ovarian cancer-related disease (*n* = 3,268, 88.1%). The 5-year OS was 27.9%, and the median OS was 36 months. The 5-year OS in high-grade serous, endometrioid, mucinous, and clear cell subtypes were 28.1, 38.6, 14.2, and 18.8%, respectively, with a median OS of 37, 40, 9, and 19 months, respectively, (*p* < 0.001) (Figure 1).

In the entire cohort, the results of multivariate analyses indicated that the histological subtype was an independent predictor for OS (Table 2). Using the high-grade serous subtype as a reference, OS was comparable for the endometrioid subtype (hazard ratio [HR] 0.915, 5% confidence interval [CI] 0.772–1.085, *p* = 0.305), while OS was significantly lower for the mucinous (HR 3.292, 95% CI 2.701–4.011, *p* < 0.001) and clear cell subtypes (HR 1.820, 95% CI 1.546–2.141, *p* < 0.001). Age, race/ethnicity, tumor grade, and metastatic site were also independent predictors of OS.

**Table 2:** Multivariate prognostic analyses.

**Variables**	**Entire cohort**			**Known the size of residual tumor**		
	**HR**	**95% CI**	***p***	**HR**	**95% CI**	***p***
**AGE (YEARS)**						
<60	1			1		
≥60	1.285	1.202–1.373	<0.001	1.066	0.904–1.258	0.448
**RACE/ETHNICITY**						
Non-hispanic white	1			1		
Non-hispanic black	1.236	1.092–1.400	0.001	1.124	0.812–1.556	0.482
Hispanic	0.941	0.844–1.049	0.272	0.834	0.638–1.090	0.184
Other	0.891	0.787–1.009	0.069	0.797	0.593–1.072	0.133
**GRADE**						
Well-differentiated	1			1		
Moderately differentiated	0.349	0.217–0.562	<0.001	—	—	0.921
Poorly/undifferentiated	0.993	0.889–1.109	0.906	1.367	0.919–2.035	0.123
Unknown	0.973	0.857–1.105	0.677	1.398	0.918–2.127	0.118
**NODAL STATUS**						
Negative	1			1		
Positive	1.055	0.982–1.134	0.143	0.967	0.813–1.151	0.706
Unknown	1.161	1.053–1.282	0.003	1.169	0.863–1.583	0.314
**METASTATIC SITE**						
Distant lymph node only (A)	1			1		
Liver parenchymal metastasis or pleural effusion with positive cytology (B)	1.169	1.038–1.317	0.010	1.071	0.796–1.440	0.652
A+B	1.233	1.058–1.438	0.007	1.263	0.876–1.820	0.211
Other	0.967	0.786–1.190	0.750	0.949	0.645–1.395	0.788
**HISTOLOGICAL SUBTYPES**						
High-grade serous	1			1		
Endometrioid	0.915	0.772–1.085	0.305	0.572	0.336–0.941	0.040
Mucinous	3.292	2.701–4.011	<0.001	3.123	1.757–5.553	<0.001
Clear cell	1.820	1.546–2.141	<0.001	1.472	1.007–2.152	0.046
**RESIDUAL TUMOR**						
No	–			1		
≤1 cm	–	–	−−	1.282	1.063–1.547	0.009
>1 cm	–	–	−−	1.634	1.341–1.991	<0.001

*CI, confidence interval; HR, hazard ratio*.

**Figure 2:**
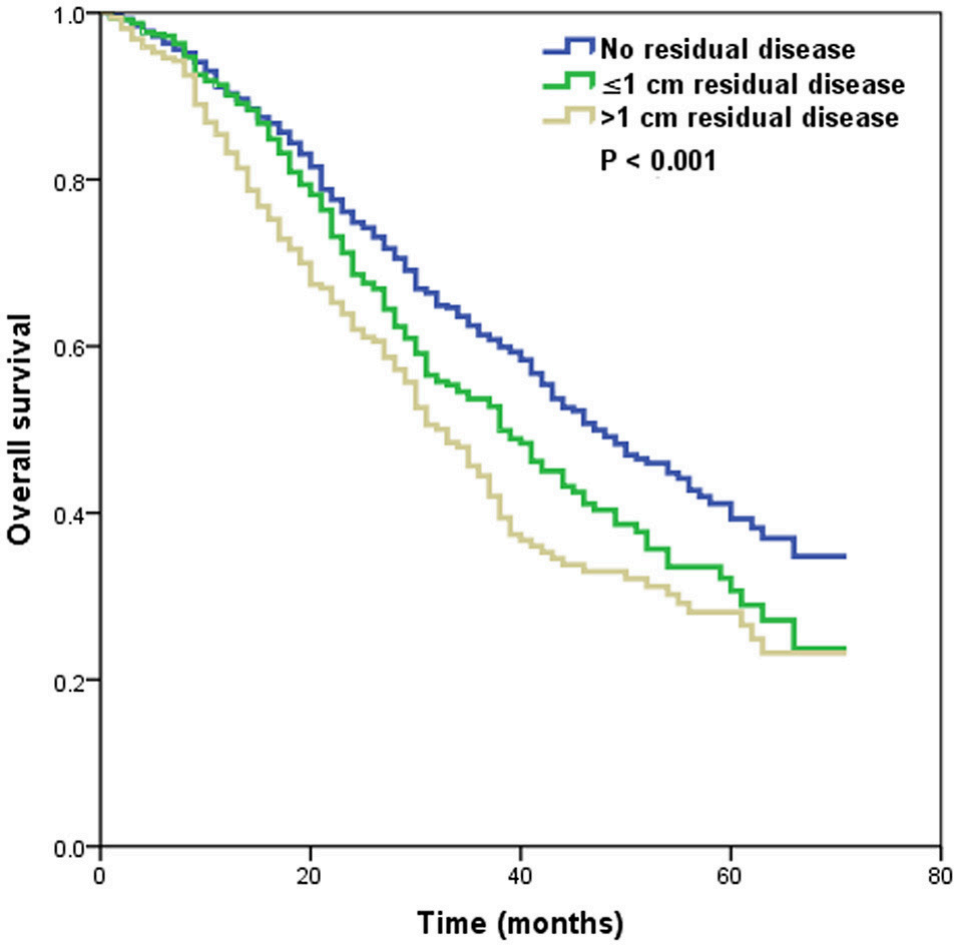
Overall survival by the size of the residual tumor.

In patients with residual tumor size available, multivariate analyses also indicated that the histological subtype was an independent predictor of OS (Table 2). Using the high-grade serous subtype as a reference, OS was better for the endometrioid subtype (HR 0.572, 95% CI 0.336–0.941, *p* = 0.040), while OS was significantly lower for the mucinous (HR 3.123, 95% CI 1.757–5.553, *p* < 0.001) and clear cell subtypes (HR 1.472, 95% CI 1.007–2.152, *p* = 0.046). In addition, the residual tumor size was also an independent prognostic factor of OS. Patients with residual tumor size ≤1 cm (HR 1.282, 95% CI 1.063–1.547, *p* = 0.009) and >1 cm (HR 1.634, 95% CI 1.341–1.991, *p* < 0.001) had poor OS compared with patients with no residual tumor. In addition, patients with >1 cm residual tumor had a poorer OS than patients with ≤1 cm residual tumor (HR 1.275, 95% CI 1.033–1.573, *p* = 0.024). The 5-year OS rates were 39.3, 30.6, and 28.1% in patients with no residual disease, ≤1 cm residual tumor, and >1 cm residual tumor, respectively, (*p* < 0.001) (Figure 2).

### The Effect of Residual Tumor Size on OS by the Specificity of Histological Subtypes

**Figure 3:**
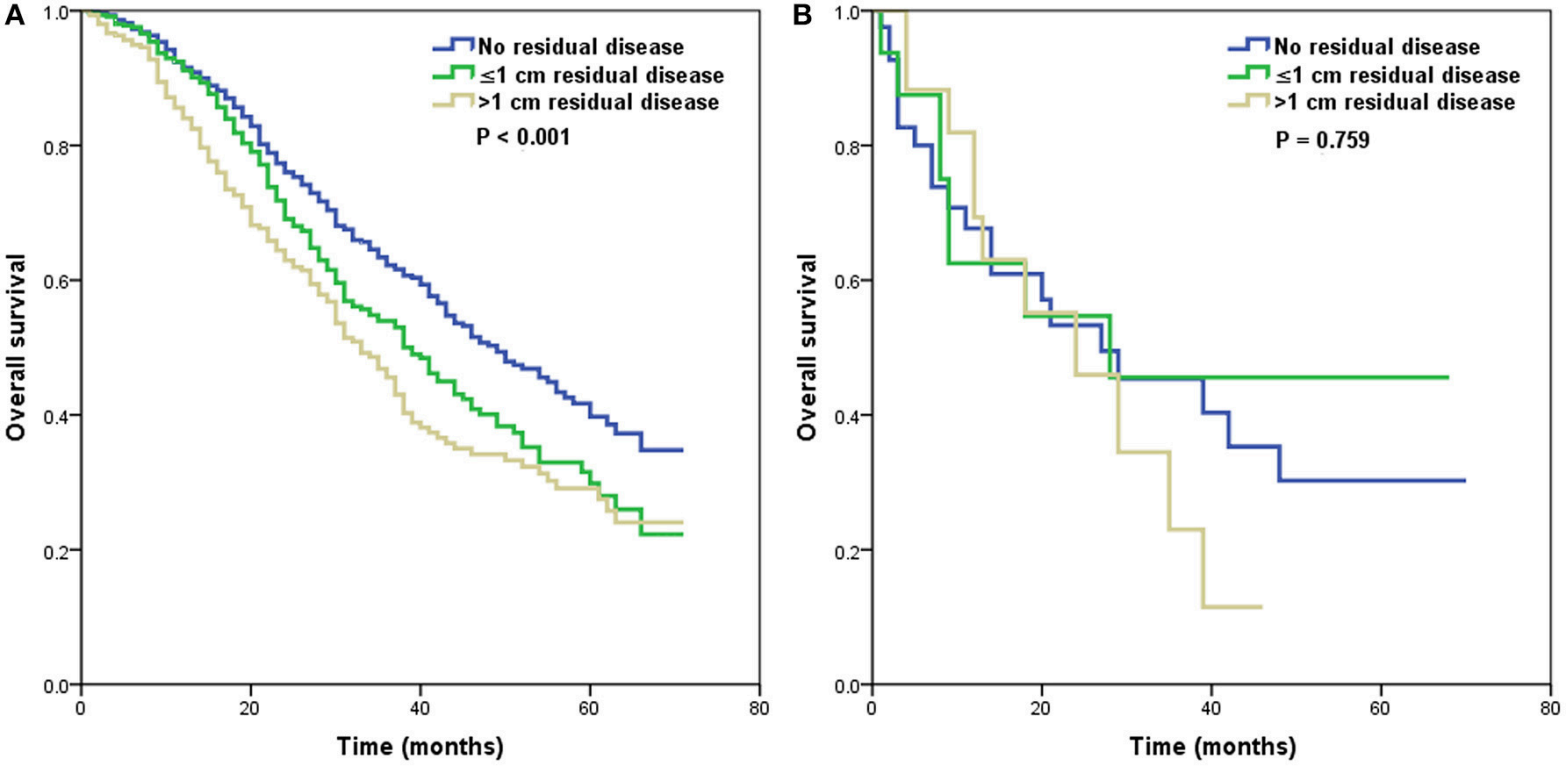
Survival curves of residual tumor size by the specificity of histological subtypes [**(A)**, high-grade serous and endometrioid subtypes; **(B)**, mucinous and clear cell subtypes].

We further analyzed the effect of the size of residual tumor on OS by the specificity of the histological subtypes (Table 3). Among the patients with residual disease, only 65, 21, and 55 patients had the endometrioid, mucinous, and clear cell subtypes, respectively. Several studies have indicated that the high-grade endometrioid subtype may be confused with serous subtype, and some high-grade endometrioid subtype were reclassified as high-grade serous carcinoma (10, 11). In addition, there were also similar outcomes between serous and endometrioid subtypes in stage IV diseasae (6, 12–14). Therefore, the high-grade serous and endometrioid subtypes were combined, and the mucinous and clear cell subtypes were combined for multivariate analyses. After adjusting for age, race/ethnicity, grade, lymph node status, and metastatic site, the size of residual tumor was an independent predictor of OS in the high-grade serous and endometrioid subtypes. Patients with residual disease ≤1 cm (HR 1.298, 95% CI 1.070–1.575, *p* = 0.008) and >1 cm (HR 1.625, 95% CI 1.323–1.995, *p* < 0.001) had poor OS compared with patients with no residual tumor. However, the residual tumor size was not a prognostic factor for OS in the mucinous and clear cell subtypes. The survival curves of residual tumor size by the specificity of the histological subtypes are shown in Figure 3 (Figure 3A, high-grade serous and endometrioid subtypes; Figure 3B, mucinous and clear cell subtypes).

**Table 3:** Multivariate prognostic analyses of the effect of residual tumor size on outcomes by the specificity of histological subtypes.

**Variables**	**HR**	**95% CI**	***p***
**HIGH-GRADE SEROUS** + **ENDOMETRIOID**			
No	1		
≤1 cm	1.298	1.070–1.575	0.008
>1 cm	1.625	1.323–1.995	<0.001
**MUCINOUS** + **CLEAR CELL**			
No	1		
≤1 cm	1.064	0.407–2.781	0.899
>1 cm	0.939	0.319–2.761	0.909

*CI, confidence interval; HR, hazard ratio*.

## Discussion

In the present study, we assessed the effect of histological subtypes on outcome of stage IV EOC. Our results indicated that the high-grade serous and endometrioid subtypes were associated with better survival, whereas patients with mucinous and clear cell subtypes were associated with a markedly higher risk of mortality.

The frequencies of histological subtypes in stage IV disease of our study (89.9, 4.2, 2.4, and 3.4% of patients had high-grade serous, endometrioid, mucinous, and clear cell subtypes, respectively) were similar to a previous SEER study including patients with stage III/IV EOC (15). However, the distribution of histological subtypes from Asia was 64.5, 11.8, 6.4, and 13.5%, respectively, (16). This is related to the marked ethnic difference of EOC between Asia and Western countries: The incidence of the clear cell subtype is ~25% of EOC in Asian, but is <10% in Western countries (17–19).

For early stage EOC, the survival of mucinous and clear cell subtypes was better than that of the serous subtype (20, 21). In an early years study (1972–1994) of advanced stage EOC, the histological subtype was not related to outcomes (22). However, the outcomes of patients with EOC were improved with the introduction of taxanes to clinical practice (23). Hosono et al. found that taxane-based chemotherapy was related to a better outcome in the serous subtype, whereas taxane-based chemotherapy was not a predictor of survival in the non-serous subtypes (24). Recent studies have confirmed that the mucinous and clear cell subtypes had worst survival outcomes compared with the serous subtype, while the endometrioid had comparable survival outcomes to the serous subtype in stage III/IV EOC (11, 25). However, a study by Ataseven et al., which included stage IV EOC, found the OS was 50.2 and 59% in the high grade serous subtype (*n* = 287) and other subtypes of EOC (*n* = 39), and the median OS was 30 and 36 months, respectively, (26).

Despite the treatment of serous carcinoma making great progress during the last 20 years, survival improvements for the mucinous and clear cell subtypes have not been observed (27). Although we could not get the information regarding the chemotherapy regimen from the SEER program, our study was wholly in the era of taxane-based chemotherapy, and our results also found that patients with serous and endometrioid subtypes had significantly better outcomes than patients with mucinous and clear cell subtypes. The 5-year OS rates were 28.1, 38.6, 14.2, and 18.8% in patients with high-grade serous, endometrioid, mucinous, and clear cell subtypes, respectively, which was similar to a study that included patients with stage III/IV EOC, in which the 5-year OS rates were 32.1, 44.7, 13.9, and 22.3%, respectively, (15). The results from Ovarian Cancer Statistics also indicated that the 5-year cause-specific survival was 26, 29, 13, and 16% for the four subtypes between 2007 and 2013, respectively, (2). Therefore, the results of our population-based analysis could represent the current survival patterns of EOC by different histological subtypes, and the treatment effect of EOC might be influenced by histology.

On the basis of randomized controlled trials, the survival outcomes in patients who received neoadjuvant chemotherapy followed by surgery were not inferior to patients who treated with primary surgery (28, 29). However, different histological subtypes may present with different responses to taxane-based and/or platinum-based chemotherapy. Patients with advanced stage mucinous carcinoma respond poorly to platinum-based chemotherapy compared with patients with other histological subtypes (20). In addition, Sugiyama et al. showed that the clear cell subtype was more related to impaired response to platinum-based chemotherapy than were serous EOC patients (11.1 vs. 72.5%) (17). Moreover, resistance to chemotherapy may not be limited to taxanes and platinum compounds (30). Therefore, it is necessary to design clinical trials to specifically explore alternative therapeutic approaches for patients with ECO with less common histologies.

In our study, the high-grade serous subtype was more prone to regional and distant lymph node metastasis, and the probability of liver parenchymal metastasis or pleural effusion was lower than in the other three subtypes. Our multivariate analysis also indicated that the survival rate of patients with liver parenchymal metastasis or pleural effusion was significantly lower than that in patients with distant lymph node metastasis only, which was similar to previous studies (7, 31). However, the results by Jamieson et al. did not show that the metastatic site, including pleural effusion, parenchymal metastases, or extra-abdominal lymph node metastases, was related to prognosis of EOC (8).

Several studies, including our results, indicated that the size of the residual tumor was related to survival outcomes of stage IV EOC (6, 8). However, Wimberger et al. found no survival difference between patients with a residual tumor ≤1 cm and those >1 cm (12). Approximately 90% of patients in our study had liver parenchymal metastasis or pleural effusion; however, these figures were 80 and 60% in the studies by Jamieson et al. (8) and Wimberger et al. (12), respectively. One explanation could be the different biological behavior in stage IV EOC, independent of the metastatic site.

Although the distribution of the size of residual disease in the four histological subtypes was not significantly different (6, 13), our results indicated a possible survival benefit of a reasonable attempt to achieve no residual disease, even in stage IV disease. However, Ayeni et al. showed that the size of the residual tumor was not related to outcomes in serous and endometrioid stage IV EOC (14). In stage III/IV mucinous and clear cell subtypes (16–32% of patients with stage IV disease), previous studies have shown better survival in patients with no residual disease, and there was no significant difference in survival between patients with residual disease ≤1 cm and >1 cm (32, 33). We only included patients with *de novo* stage IV EOC in this study, and patients with high-grade serous and endometrioid subtypes had significantly better outcomes when they had no residual tumor; however, the residual disease status was not related to the outcomes of the mucinous and clear cell subtypes. Owing to the limited number of patients with mucinous (*n* = 21) and clear cell subtypes (*n* = 55) in the cohort with residual tumor status available. It is difficult to draw a conclusion regarding to the aggressive surgical procedures in stage IV mucinous and clear cell EOC. As the higher disease burden and surgical complexity of stage IV patients, postoperative morbidity maybe significantly increased in patients who receive aggressive complex surgical cytoreduction (34, 35). More studies are needed to investigate the role of aggressive surgical procedures in patients with different biological nature of EOC subtypes.

We recognize that there were several limitations of our study. First, as a retrospective study, potential biases were unavoidable. Second, the histological subtypes might be somewhat inconsistent with current practice because some high-grade endometrioid tumors may be reclassified as high-grade serous tumors (10). However, we did not find any survival differences among patients with serous and endometrial subtypes in the entire cohort. Third, treatment information was only available for the receipt of surgery and chemotherapy; however, the sequence of treatments, chemotherapy drugs, cycles of chemotherapy, targeted therapy, and type of cytoreductive surgery were not available. In addition, the performance status, comorbidities, patterns of disease recurrence, and treatment after disease recurrence were not recorded in SEER. A final limitation of the present study was the limited number of patients with endometrioid, mucinous, and clear cell subtypes.

## Conclusion

In conclusion, our study indicates a markedly higher risk of mortality among patients with mucinous and clear cell subtypes, while patients with high-grade serous and endometrioid subtypes had better survival. However, in this subgroup of patients, the net survival remains poor overall. Aggressive radical surgery to achieve no residual tumor would improve survival in high-grade serous and endometrioid carcinoma. More studies are needed to assess the value of aggressive radical surgery in patients with mucinous and clear cell EOC. In addition, more therapeutics targeting the unique molecular features of each histological subtype are needed.

## Author Contributions

JZ and S-GW are lead authors who participated in data collection, manuscript drafting, table and figure creation, and manuscript revision. JW, J-YS, and Z-YH are senior authors who aided in drafting the manuscript and manuscript revision. W-WZ and XJ are the corresponding authors who initially developed the concept and revised the manuscript. All authors read and approved the final manuscript.

### Conflict of Interest Statement

The authors declare that the research was conducted in the absence of any commercial or financial relationships that could be construed as a potential conflict of interest.

## Footnotes

^1^ Surveillance, Epidemiology, and End Results (SEER) Program (www.seer.cancer.gov) SEER^*^Stat Database: Incidence - SEER 18 Regs Custom Data (with additional treatment fields), Nov 2017 Sub (1973-2015 varying) - Linked To County Attributes - Total U.S., 1969-2016 Counties, National Cancer Institute, DCCPS, Surveillance Research Program, released April 2018, based on the November 2017 submission.
